# Transcriptomic Characterization of C57BL/6 Mouse Embryonic Stem Cell Differentiation and Its Modulation by Developmental Toxicants

**DOI:** 10.1371/journal.pone.0108510

**Published:** 2014-09-23

**Authors:** Xiugong Gao, Jeffrey J. Yourick, Robert L. Sprando

**Affiliations:** Division of Toxicology, Office of Applied Research and Safety Assessment, Center for Food Safety and Applied Nutrition, U.S. Food and Drug Administration, Laurel, Maryland, United States of America; Michigan State University, United States of America

## Abstract

The Tox21 program calls for transforming toxicology testing from traditional *in vivo* tests to less expensive and higher throughput *in vitro* methods. In developmental toxicology, a spectrum of alternative methods including cell line based tests has been developed. In particular, embryonic stem cells (ESCs) have received widespread attention as a promising alternative model for developmental toxicity assessment. Here, we characterized gene expression changes during mouse ESC differentiation and their modulation by developmental toxicants. C57BL/6 ESCs were allowed to differentiate spontaneously and RNA of vehicle controls was collected at 0, 24, 48, 72, 96, 120 and 168 h after embryoid body (EB) formation; RNA of compound-exposed EBs were collected at 24 h. Samples were hybridized to Affymetrix Mouse Gene 2.0 ST Array; using stringent cut-off criteria of Bonferroni-adjusted *p*<0.05 and fold change >2.0, a total of 1996 genes were found differentially expressed among the vehicle controls at different time points. Gene ontology (GO) analysis showed these regulated genes were mostly involved in differentiation-related processes such as development, morphogenesis, metabolism, cell differentiation, cell organization and biogenesis, embryonic development, and reproduction. Biomarkers of all three germ layers or of their derivative early cell types were identified in the gene list. Principal component analysis (PCA) based on these genes showed that the unexposed vehicle controls appeared in chronological order in the PCA plot, and formed a differentiation track when connected. Cultures exposed to thalidomide, monobutyl phthalate, or valproic acid deviated significantly from the differentiation track, manifesting the capacity of the differentiation track to identify the modulating effects of diverse developmental toxicants. The differentiation track defined in this study may be further exploited as a baseline for developmental toxicity testing, with compounds causing significant deviation from the differentiation track being predicted as potential developmental toxicants.

## Introduction

Toxicity testing has traditionally relied on animal models which are costly, time consuming and low throughput. Moreover, it often causes pain and stress to, and frequently involves the sacrifice of, large numbers of laboratory animals. This is especially true for reproductive and developmental toxicity testing [1]. With the EU chemicals regulation protocol REACH [2] in force, it has been estimated that over 70–80% of all animals used for safety testing would be used for examining reproductive and developmental toxicity [3], [4]. Under such circumstances, the Tox21 program [5] partnered by several US Federal agencies calls for transforming toxicology testing from traditional *in vivo* tests to less expensive and higher throughput *in vitro* methods to prioritize compounds for further study, identify mechanisms of action and ultimately develop predictive models for adverse health effects in humans. In support of the program, the US FDA is developing alternative models for safety assessment of foods, dietary supplements and cosmetics.

Over the last three decades, multiple alternative *in vitro* or nonmammalian *in vivo* models for developmental toxicity screening has been developed. Examples of *in vivo* nonmammalian models include invertebrates such as the nematode (*Caenorhabditis elegans*) and fruit fly (*Drosophila melanogaster*), and vertebrates such as the frog (*Xenopus laevis*) and zebrafish (*Danio rerio*) [6]. Alternative *in vitro* test systems utilize organ-, embryo-, or cell-cultures and include the limb bud micromass (MM) [7], the rat postimplantation whole embryo culture (WEC) [8], and the mouse embryonic stem cell test (EST) [9].

Embryonic stem cells (ESCs) have gained considerable interest for their use in developmental toxicity testing due to their fundamental attributes of unlimited expansion and pluripotency [10]. The EST was developed by Spielmann and his group as an *in vitro* model for the screening of embryotoxicity based on the interference of chemicals with the differentiation of mouse embryonic stem cells (mESCs) into beating cardiomyocyte foci in culture [11]. A blastocyst-derived permanent mESC cell line (D3) derived from mouse 129 strains was used in the test [12]. The test was successfully validated by the European Center for the Validation of Alternative Methods (ECECVAM) [13]. However, in subsequent testing using new sets of chemicals and pharmaceutical compounds, the EST performed well below the 78% accuracy expected from the validation study [14]. This could be partly attributed to the prediction model used, which was purely mathematical with its biological relevance unclear [15]. In addition, the applicability domain of the assay, which is currently limited to substances that do not require metabolic conversion and act in early embryonic development [14], is not adequate for assessing diverse classes of developmental toxicants. Employing multiple endpoints in the test model to replace or supplement the current subjective single-lineage readout (scoring of contracting cardiomyocyte outgrowths) would lead to an improved definition of the applicability domain and the associated predictive capacity, thus increasing the usefulness of the EST in developmental toxicity testing.

Advances in genomics technologies have enabled the measurement of tens of thousands of endpoints in a single assay, such as transcriptomics that evaluates genome-wide gene expression changes. Genomic profiling in toxicity studies, commonly referred to as toxicogenomics, can be used to delineate mechanisms of action of potential human and environmental toxicants, and to identify biomarkers that may improve the prediction of specific toxic effects. The identified biomarkers may also be used to discriminate or categorize compound classes, as while each compound may have its distinct gene expression signatures, compounds of a common chemical class will likely affect similar biological processes, thus inducing reproducible gene-expression responses with a recognizable overlap [16]. In addition, assay endpoints in the form of gene expression profiles could be detected earlier, or at lower doses, than classical biological endpoints [16], such as the morphological scoring and cytotoxicity assays used in the EST.

In recent years, a series of studies have been carried out by Piersma and colleagues in an effort to implement toxicogenomics into the EST to improve its application domain and predictability (for a review, see [17]). These studies, using gene sets within the biological domain of the differentiation processes present in the assay, have shown promising results in determining the predictive capacity of the EST [18], [19]. However, further studies are needed to expand this line of research in order to improve predictability on the basis of a well-defined applicability domain.

Historically, mESCs derived from the 129 mouse strains have been widely used in biomedical research, especially in generating genetically altered mouse models, by virtue of their high targeting efficiency and proven germ line transmission. In recent years, germline-competent mESCs have also been derived from other strains such as C57BL/6 [20], [21], which would support broader use of mESCs in biomedical research. However, literature on using C57BL/6 mESCs for toxicological studies is virtually blank, except that Hubbard *et al.* [22] used neuronal cultures derived from a C57BL/6 cell line to study its functional responses to neurotropic toxins.

Herein, as part of an effort to develop an ESC-based alternative model for the assessment of developmental toxicity, we characterized gene expression changes during the differentiation of a C57BL/6-derived mESC cell line. We showed that the overall gene expression profile of the C57BL/6 mESCs gradually changed during the course of differentiation, which chronologically formed a differentiation track. We further demonstrated that the differentiation track was able to identify the modulating effects of three developmental toxicants: thalidomide (THD), monobutyl phthalate (MBP), and valproic acid (VPA). These chemicals were selected because they are considered to have diverse mechanisms of developmental toxicity. THD is the notorious teratogen that causes congenital limb malformation in human and some animal species, but not in mouse [23]. MBP is the embryotoxic metabolite of a group of industrial chemicals called phthalates or phthalate esters, which showed a variety of toxic effects in animal studies, in particular on reproduction and development [24]. VPA is another well-known teratogen that causes neural tube defects (NTD) in children affected [25]. We propose the differentiation track defined in this study be further exploited as a baseline for developmental toxicity testing, with compounds causing significant deviation from the differentiation track being predicted as potential developmental toxicants.

## Materials and Methods

### Materials

All chemicals were of molecular biology grade and were obtained from Sigma-Aldrich (St. Louis, MO) unless otherwise stated.

### Pluripotent Mouse Embryonic Stem Cell Culture

Pluripotent ESGRO Complete Adapted C57BL/6 mouse ESCs, which have been pre-adapted to serum-free and feeder-free culture condition, were obtained from EMD Millipore (Billerica, MA) at passage 12 (with 80% normal male mouse karyotype). The cells were seeded on 0.1% gelatin-coated flasks, and maintained at 37°C in a 5% CO_2_ humidified incubator at standard densities (*i.e.*, between 5×10^4^/cm^2^ and 5×10^5^/cm^2^) in ESGRO Complete Plus Clonal Grade Medium (EMD Millipore). The medium contains leukemia inhibitory factor (LIF), bone morphogenic protein 4 (BMP-4), and a glycogen synthase kinase-3b inhibitor (GSK3b-I) to help maintain pluripotency and self-renewal of the ESCs. Cells were passaged every 2–3 days (when reaching 60% confluence) with ESGRO Complete Accutase (EMD Millipore) at about 1∶6 ratio. C57BL/6 ESCs maintain a stable karyotype under the above passaging condition. The cells used for differentiation and gene expression studies were at passage 18.

### Cell Differentiation through Embryoid Body Formation

Induction of differentiation was achieved through embryoid body (EB) formation via hanging drop culture following a procedure adapted from De Smedt *et al.* [26]. In brief, stem cells were thawed and a suspension was prepared at a concentration of 3.75×10^4^ cells/ml in ESGRO Complete Basal Medium (EMD Millipore), which does not contain LIP, BMP-4, or GSK3b-I. About 50 drops (each of 20 µl) of the cell suspension were placed onto the inner side of the lid of a 10-cm Petri dish filled with 5 ml phosphate buffered saline (PBS; EMD Millipore) and incubated at 37°C and 5% CO_2_ in a humidified atmosphere. After 3 days, EBs formed in the hanging drops (Ø330–350 µm) were subsequently transferred into 6-cm bacteriological Petri dishes (Becton Dickinson Labware, Franklin Lakes, NJ) and were further cultivated for 2 days. On day 5, EBs were plated one per well into 24-well tissue culture plates (Thermo Scientific Nunc, Roskilde, Denmark). During further development of the attached EBs, cells of endodermal, ectodermal and mesodermal origin were obtained in the outgrowths. In EST, differentiation was determined by microscopic inspection of contracting cardiomyocytes in the EB outgrowths on day 10.

### Exposure to Test Compounds and RNA Isolation

**Figure 1:**
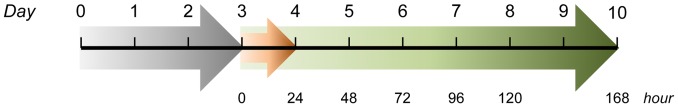
Schematic representation of the experimental procedure. The grey arrow covers the embryoid body (EB) formation stage. Hanging drops were set up on day 0 and EBs formed on day 3. The green arrow depicts the ESC differentiation period, starting from day 3 and ending on day 10. Compound exposure is shown by the orange arrow, which lasted only 24 h (from day 3 to day 4). The numbers on the top are days covering the whole process, while the numbers at the bottom are the time points in hours during ESC differentiation (after EB formation) that were sampled in the present study.

ESC differentiation cultures were exposed from the EB stage at day 3 onwards to 0.25 mM thalidomide (THD), 2.0 mM monobutyl phthalate (MBP), 1.0 mM valproic acid (VPA), or vehicle (0.25% DMSO). Preliminary results showed that DMSO at 0.25% (v/v) had no significant effect on gene expression during C57BL/6 ESC differentiation under the condition used in the study (data not shown). The concentrations used for the test compounds (THD, MBP, and VPA) were previously used in similar toxicogenomic studies with mESCs [27], [28]. Vehicle control cultures were collected at 0, 24, 48, 72, 96, 120 and 168 h after EB formation (culture days 3, 4, 5, 6 7, 8 and 10). Compound-exposed cultures were collected at 24 h (culture day 4) (Fig. 1). This time point was chosen as it is amenable to high-throughput screening (HTS). Three biological replicates were used for each condition. Treatment with compounds did not affect EB sizes (data not shown). EBs were lysed in RLT buffer (Qiagen; Valencia, CA) supplemented with β-mercaptoethanol, homogenized by QIAshredder (Qiagen), and kept in a −80°C freezer until further processing. Total RNA was isolated on the EZ1 Advanced XL (Qiagen) automated RNA purification instrument using the EZ1 RNA Cell Mini Kit (Qiagen) following the manufacturer's protocol, including an on-column DNase digestion. RNA concentration and purity (260/280 ratio) were measured with the NanoDrop 2000UV-Vis spectrophotometer (NanoDrop Products, Wilmington, DE). Integrity of RNA samples was assessed by the Agilent 2100 Bioanalyzer (Santa Clara, CA) with the RNA 6000 Nano Reagent Kit from the same manufacturer.

### RNA Processing and Microarray Experiment

The total RNA samples were preprocessed for hybridization to Mouse Gene 2.0 ST Array (Affymetrix, Santa Clara, CA) using the GeneChip WT PLUS Reagent Kit (Affymetrix) following the manufacturer's protocol. In brief, 50 ng of total RNA was used to generate first strand cDNA using reverse transcriptase and primers containing a T7 promoter sequence.The single-stranded cDNA was then converted to double-stranded cDNA by using DNA polymerase and RNase H to simultaneously degrade the RNA and synthesize second-strand cDNA. Complimentary RNA (cRNA) was synthesized and amplified by *in vitro* transcription (IVT) of the second-stranded cDNA template using T7 RNA polymerase. Subsequently, sense-strand cDNA was synthesized by the reverse transcription of cRNA with incorporated deoxyuridine triphosphate (dUTP). Purified, sense-strand cDNA was fragmented by uracil-DNA glycosylase (UDG) and apurinic/apyrimidinic endonuclease 1 (APE 1) at the unnatural dUTP residues and labeled by terminal deoxynucleotidyl transferase (TdT) using the Affymetrix proprietary DNA Labeling Reagent that is covalently linked to biotin. Subsequent hybridization, wash, and staining were carried out using the Affymetrix GeneChip Hybridization, Wash, and Stain Kit and the manufacturer's protocols were followed. Briefly, each fragmented and labeled sense-strand cDNA target sample (approximately 3.5 µg) was individually hybridized to a GeneChip Mouse Gene 2.0 ST Array at 45°C for 16 h in Affymetrix GeneChip Hybridization Oven 645. After hybridization, the array chips were stained and washed using an Affymetrix Fluidics Station 450. The chips were then scanned on Affymetrix GeneChip Scanner 3000 7G and the image (.DAT) files were preprocessed using the Affymetrix GeneChip Command Console (AGCC) software v.4.0 to generate cell intensity (.CEL) files. Prior to data analysis, all arrays referred to in this study were assessed for data quality using the Affymetrix Expression Console software v.1.3 and all quality assessment metrics (including spike-in controls during target preparation and hybridization) were found within boundaries. The data set has been deposited in Gene Expression Omnibus (GEO; http://www.ncbi.nlm.nih.gov/geo/) of the National Center for Biotechnology Information with accession number GSE60174.

### Data Processing and Statistical Analysis

The values of individual probes belonging to one probe set in .CEL files were summarized using the robust multi-array average (RMA) algorithm [29] embedded in the Expression Console software v.1.3 (Affymetrix), which comprises of convolution background correction, quantile normalization, and median polish summarization. Downstream data analysis was carried out primarily using the US FDA's ArrayTrack software system [30], [31]. Normalized data for all samples were first analyzed by unsupervised principal component analysis (PCA) [32] and hierarchical cluster analysis (HCA) [33], to identify patterns in the dataset and highlight similarities and differences among the samples. Subsequently, differentially expressed genes (DEGs) were selected using one-way analysis of variance (ANOVA) or pairwise *t*-tests. The fold change (FC) of every gene, together with their corresponding *p*-value, was used for selection of DEGs with cutoff values indicated in the text.

### Gene Ontology and Pathway Analysis

Genes whose expression was significantly regulated were subjected to gene ontology (GO) and pathway analysis using the Database for Annotation, Visualization, and Integrated Discovery (DAVID) [34] to find overrepresentations of GO terms in the biological process (BP) category at all levels (GOTERM_BP_ALL) and KEGG pathways. As background, the *Mus musculus* (mouse) whole genome was used. Statistical enrichment was determined through a modified Fisher's exact test (*p*<0.05) and count threshold >4 for GO terms and a modified Fisher's exact test (*p*<0.01) and count threshold >10 for KEGG pathways. The statistically enriched GO terms were grouped and counted after classification according to GO Slim using the freely available web tool CateGOrizer [35]. The text-mining tool Anni [36] was also used to explore matching concept profiles of gene clusters with concept profiles of biological processes in the GO database. The program calculates the overall matching score, the cohesion score, between each gene of the gene cluster with the concept profiles of the GO biological processes. The concepts with the highest sum of cohesion scores are considered the predominant functions of the gene cluster.

## Results

### Overall Gene Expression Changes and Associated Biological Functions

**Figure 2:**
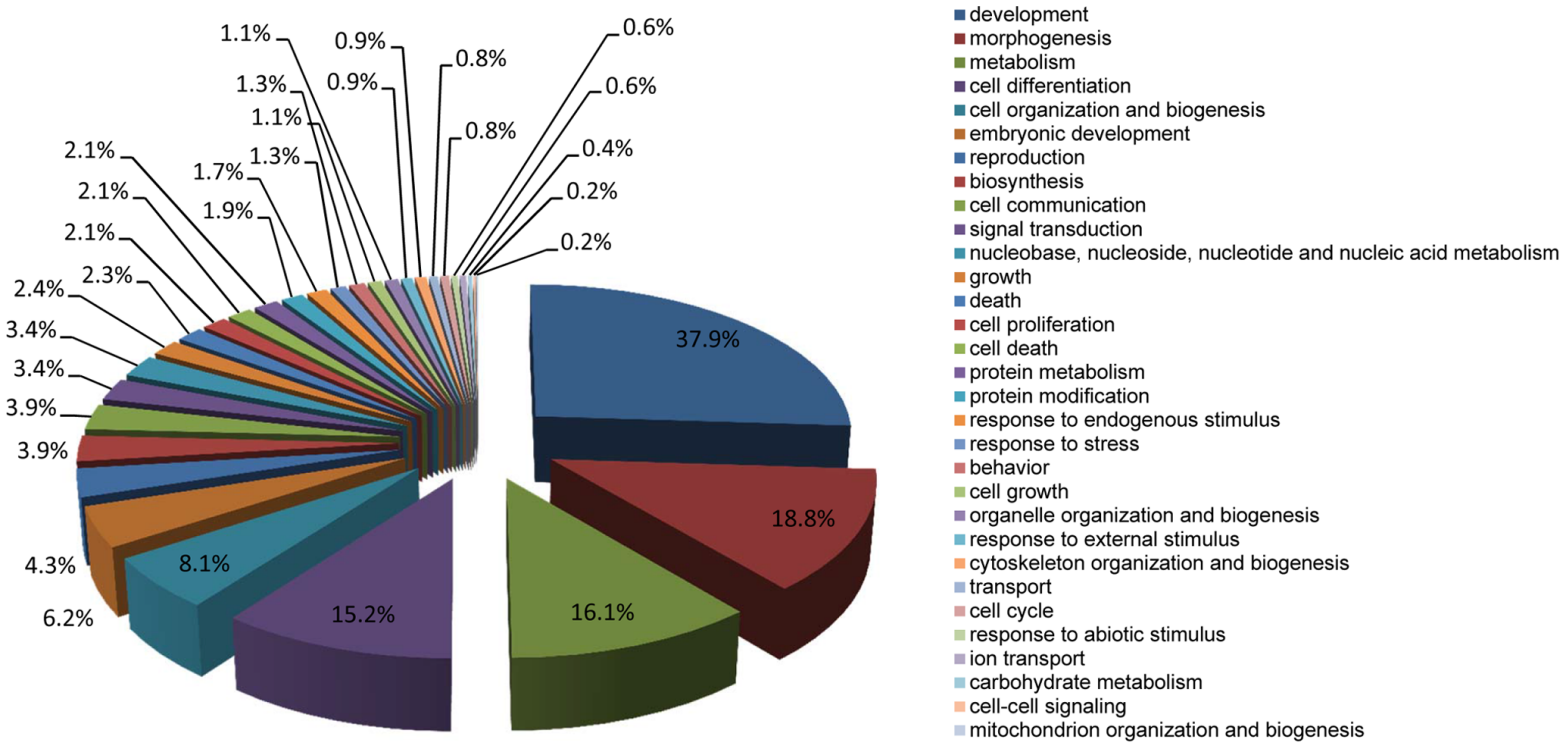
Distribution of enriched GO terms according to GO slim for the 1996 DEGs identified during ESC differentiation. The percentage indicates the number of GO terms in each class as a percentage of the total number of unique GO terms (533) enriched by the DEGs.

Gene expression data of control samples at 0, 24, 48, 72, 96, 120 and 168 h after EB formation were compared by one-way ANOVA. Using stringent cut-off criteria of Bonferroni-adjusted *p*<0.05 and FC>2.0, a total of 1996 genes (referred henceforth as “1996 DEGs”) were found differentially expressed across the different time points, with 1229 upregulated and 767 downregulated along the time course. Out of these genes, 1675 were mapped to the DAVID database as annotated genes (Table S1). GO analysis revealed 1268 (76.2%) of these annotated genes were enriched in 541 (533 unique) GO terms in the BP category at all levels (Table S2). Using the CateGOrizer tool, these GO terms were grouped into 31 classes within the pre-defined set of parent/ancestor GO terms (Fig. 2), and were found mostly involved in differentiation-related processes, including development (37.9%), morphogenesis (18.8%), metabolism (16.1%), cell differentiation (15.2%), cell organization and biogenesis (8.1%), and embryonic development (6.2%).

The KEGG pathways enriched in the 1996 DEGs, as analyzed by DAVID, are listed in Table 1. Among them were several signaling pathways involved in ESC proliferation, differentiation, and tissue/organ development, including the mitogen-activated protein kinase (MAPK) signaling pathway, the Wnt signaling pathway, the Hedgehog signaling pathway, and the transforming growth factor (TGF)-beta signaling pathway. Also in the list were the focal adhesion pathway, the extracellular matrix (ECM)-receptor interaction pathway, the adherens junction pathway, and the gap junction pathway. These pathways are involved in cell-matrix adhesion, cell-receptor interaction, and cell-cell communication, all playing essential roles in cell proliferation and/or differentiation. The p53 signaling pathway, important in cell cycle regulation, was also enriched in the DEGs. So were some pathways involved in specific tissue/organ development, such as the axon guidance pathway and the melanogenesis pathway.

**Table 1:** List of KEGG pathways enriched by the 1996 DEGs during ESC differentiation.

Term	Pathway	Count*	%**	*p*-value	Fold enrichment
mmu00330	Arginine and proline metabolism	12	0.72	6.51E-03	2.51
mmu04115	p53 signaling pathway	15	0.90	2.83E-03	2.41
mmu04010	MAPK signaling pathway	37	2.22	7.32E-03	1.55
mmu04310	Wnt signaling pathway	25	1.50	3.30E-03	1.86
mmu04340	Hedgehog signaling pathway	13	0.78	2.51E-03	2.67
mmu04350	TGF-beta signaling pathway	25	1.50	3.56E-07	3.19
mmu04510	Focal adhesion	45	2.70	7.93E-09	2.52
mmu04512	ECM-receptor interaction	27	1.62	6.23E-09	3.61
mmu04520	Adherens junction	15	0.90	7.02E-03	2.19
mmu04540	Gap junction	17	1.02	3.69E-03	2.19
mmu04360	Axon guidance	36	2.16	1.79E-09	3.05
mmu04916	Melanogenesis	18	1.08	7.27E-03	2.00
mmu05414	Dilated cardiomyopathy	24	1.44	4.05E-06	2.90
mmu05410	Hypertrophic cardiomyopathy (HCM)	22	1.32	1.06E-05	2.91
mmu05412	Arrhythmogenic right ventricular cardiomyopathy (ARVC)	16	0.96	2.36E-03	2.37
mmu05200	Pathways in cancer	71	4.27	6.74E-13	2.44
mmu05210	Colorectal cancer	28	1.68	2.99E-09	3.61
mmu05212	Pancreatic cancer	16	0.96	1.54E-03	2.47
mmu05213	Endometrial cancer	13	0.78	1.79E-03	2.77
mmu05215	Prostate cancer	23	1.38	9.72E-06	2.84
mmu05217	Basal cell carcinoma	18	1.08	3.39E-06	3.63
mmu05218	Melanoma	15	0.90	3.73E-03	2.34
mmu05220	Chronic myeloid leukemia	17	1.02	9.61E-04	2.48
mmu05221	Acute myeloid leukemia	14	0.84	1.31E-03	2.73
mmu05222	Small cell lung cancer	20	1.20	1.44E-04	2.61

DAVID was used for the analysis using the 1675 annotated genes from the 1996 DEGs identified during ESC differentiation (see text and Table S1 for details).

*Number of DEGs involved in the pathway.

**Number of DEGs involved in the pathway as a percentage of the total number of annotated genes (1675).

**Figure 3:**
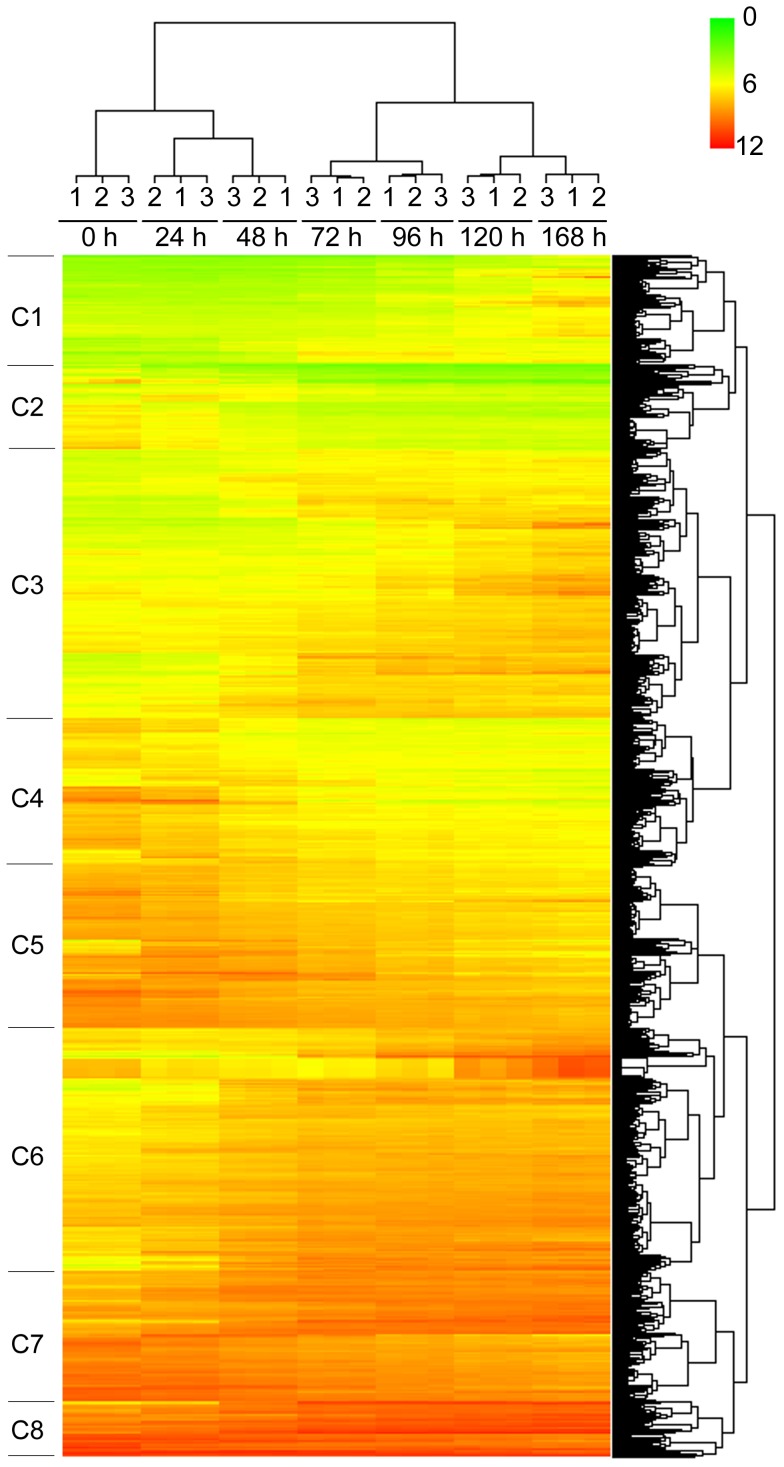
Hierarchical clustering of the 1996 DEGs. Each row represents a probe set and each column represents a sample. The expression data are presented as log_2_ values with color schemes shown on the top-right corner. Broadly eight clusters were identified (C1 to C8) each with a distinct gene expression profile in terms of their expression dynamics with time.

The hierarchical clustering of the 1996 DEGs is shown in Fig. 3. Broadly eight clusters were identified, each with a distinct gene expression profile in terms of their expression dynamics with time. The predominant functions of each of the eight clusters were determined by Anni 2.1 text-mining analysis, and these are presented in Table 2.

**Table 2:** Compilation of predominant functions for each of the eight clusters (C1–C8) defined by HCA.

	C1	C2	C3	C4	C5	C6	C7	C8	Σ
Antigen presentation				+					1
Arachodonic acid metabolism		+							1
Axon guidance	+		+			+			3
Cell adhesion	+								1
Cell cycle							+		1
Cell cycle arrest							+		1
Cell cycle checkpoint					+				1
Cell movement						+			1
Chondrocyte differentiation			+						1
Chondrogenesis			+						1
Cyclin-dependent protein kinase inhibitor activity							+	+	2
DNA methylation		+						+	2
Epithelial to mesenchymal transition						+		+	2
Gastrulation		+		+					2
Heart development			+						1
Leukotriene biosynthesis		+							1
Mesoderm formation				+					1
Mitotic cell cycle							+	+	2
Neurogenesis	+								1
Paraxial mesoderm formation		+							1
Phosphorylation	+		+	+		+			4
Protein tagging activity					+				1
Ribosome biogenesis and assembly					+				1
rRNA processing					+				1
Somitogenesis				+					1
Tumor suppressor activity	+				+	+	+	+	5

The top 5 functions as determined by Anni 2.1 for each of the 8 clusters (C1–C8) defined in HCA and shown in Fig. 3 are compiled here. A “+” sign means a cluster indicated by the title of the column has the function shown as the title of the row. The last column “Σ” indicates the total number of clusters sharing the same function (of that row).

**Figure 4:**
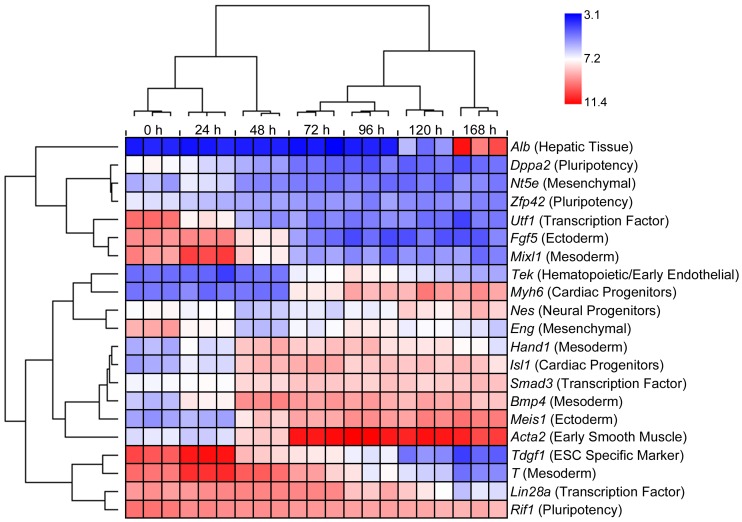
Hierarchical clustering of 21 marker genes important for ESC differentiation. Each row represents a marker gene and each column represents a sample. The expression data are presented as log_2_ values with color schemes shown on the top-right corner. The functional category of each marker gene is shown in the parentheses following the gene name.

A score of marker genes important for ESC differentiation were identified in the 1996 DEGs. These genes including an ESC specific marker (*Tdgf1*) and transcriptional factors (*Lin28a*, *Smad3*, and *Utf1*), pluripotency markers (*Dppa2*, *Rif1*, and *Zfp42*), germ layer markers (*Fgf5* and *Meis1* for ectoderm, and *Hand1*, *Mixl1*, *Bmp4* and *T* for mesoderm), and markers for early cell types including those for neural progenitors (*Nes*), cardiac progenitors (*Isl1* and *Myh6*), early smooth muscle cells (*Acta2*), hematopoietic stem cells/early endothelial cells (*Tek*), mesenchymal stem cells (*Eng* and *Nt5e*), and hepatic tissue (*Alb*). The heat map of these genes is shown in Fig. 4.

### Dynamics of Gene Expression Changes during ESC Differentiation

To further explore the dynamic nature of gene expression changes during ESC differentiation, the DEGs at each time point as compared with the starting point (0 h after EB formation) were analyzed by pairwise *t*-test (*p*<0.05, FC>2.0) and the numbers are listed in Table 3. There were generally more upregulated genes than downregulated genes at each time point except for 24 h, where the number of downregulated genes was slightly higher. The number of total DEGs gradually increased with time from 24 h to 72 h, then remained stable up to 120 h, and increased again at 168 h. The numbers of upregulated and those of downregulated genes follow the same trend.

**Table 3:** Number of DEGs at each time point during ESC differentiation as compared with the starting point (0 h after EB formation).

Time point (h)	Upregulated	Downregulated	All
24	178	208	386
48	549	425	974
72	817	610	1427
96	841	538	1379
120	901	666	1567
168	1255	867	2122

Pairwise *t*-tests were used for the analysis. The DEGs were selected using *p*<0.05 and FC>2.0.

**Figure 5:**
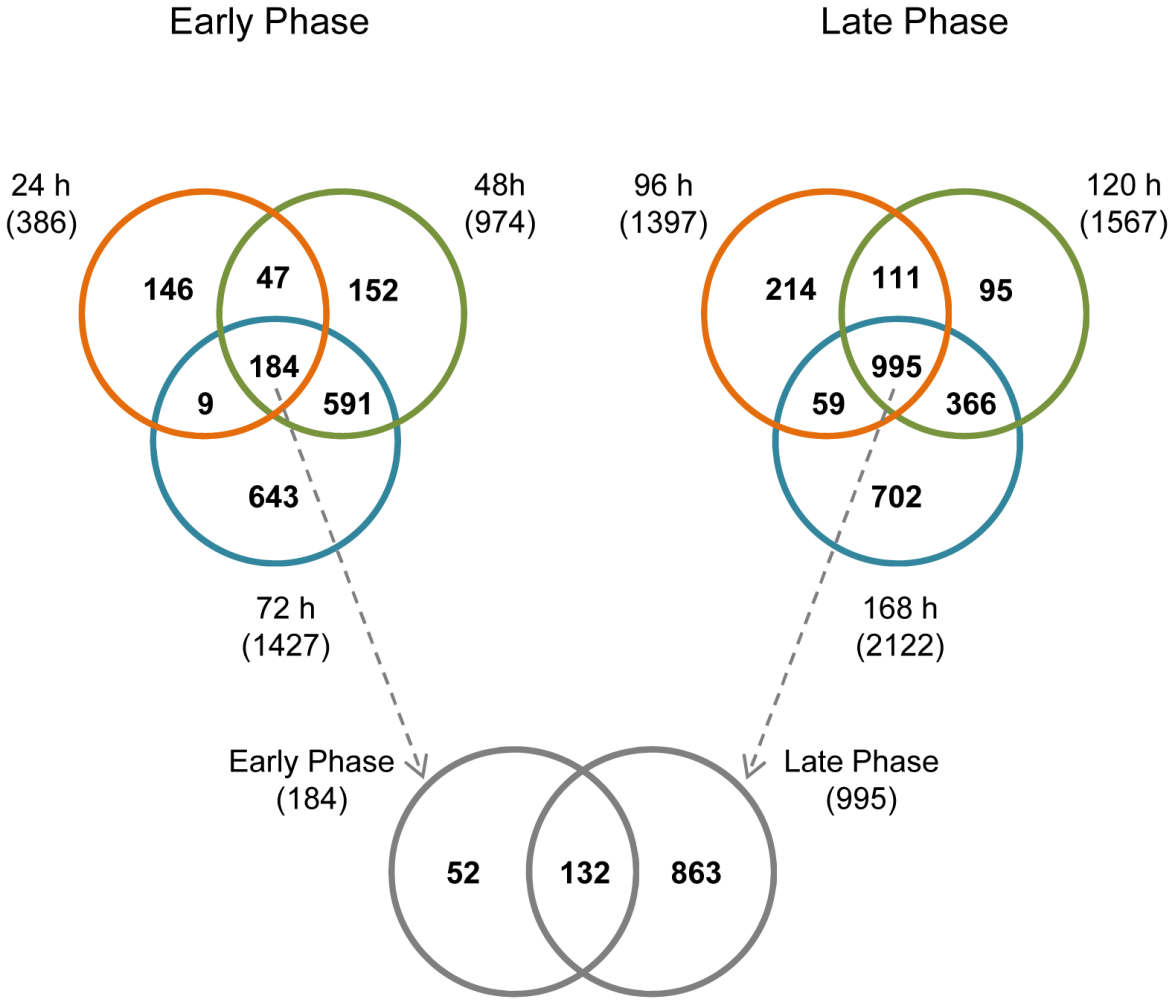
Venn diagrams showing overlap of DEGs between different time points. The total number of DEGs at each time point is included in the parentheses under the time. The overlapping of DEGs in the Venn diagrams took into consideration of fold change direction.

The overlapping of all DEGs (both upregulated and downregulated) at each time point is shown in Fig. 5. In the early differentiation phase (24 h, 48 h, and 72 h), only a small portion of DEGs (184) was shared by all the three time points, compared to the total number of DEGs (386, 974, and 1427) at each time point. In the later phase (96 h, 120 h, and 168 h), a larger portion was shared by all the three time points (995 compared to 1379, 1567 and 2122). Between the 184 common DEGs of the early phase and 995 of the later phase, a total of 132 DEGs was shared; out which 96 were mapped to the DAVID database as annotated genes (Table S3). Therefore, 96 annotated genes (referred henceforth as “96 DEGs”) were differentially expressed throughout the differentiation process covered in this study.

**Figure 6:**
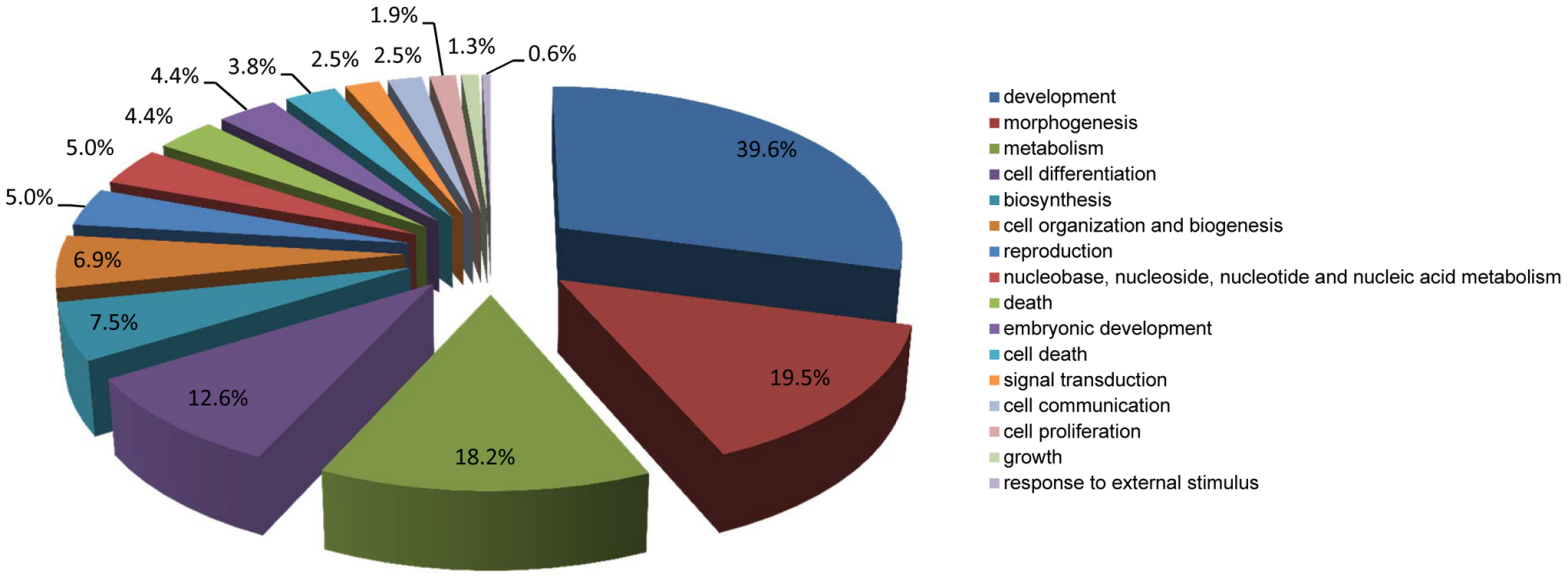
Distribution of enriched GO terms according to GO slim for the 96 DEGs identified throughout ESC differentiation. The percentage indicates the number of GO terms in each class as a percentage of the total number of unique GO terms (159) enriched by the DEGs.

GO analysis of the 96 DEGs revealed 81 (84.4%) of them were enriched in 162 (159 unique) GO terms in the BP category at all levels (Table S4). These GO terms were grouped into 16 classes (Fig. 6) by CateGOrizer. Similar to the previous findings, on top of the list were development (39.6%), morphogenesis (19.5%), metabolism (18.2%), cell differentiation (12.6%), biosynthesis (7.5%), and cell organization and biogenesis (6.9%).

### ESC Differentiation Track

**Figure 7:**
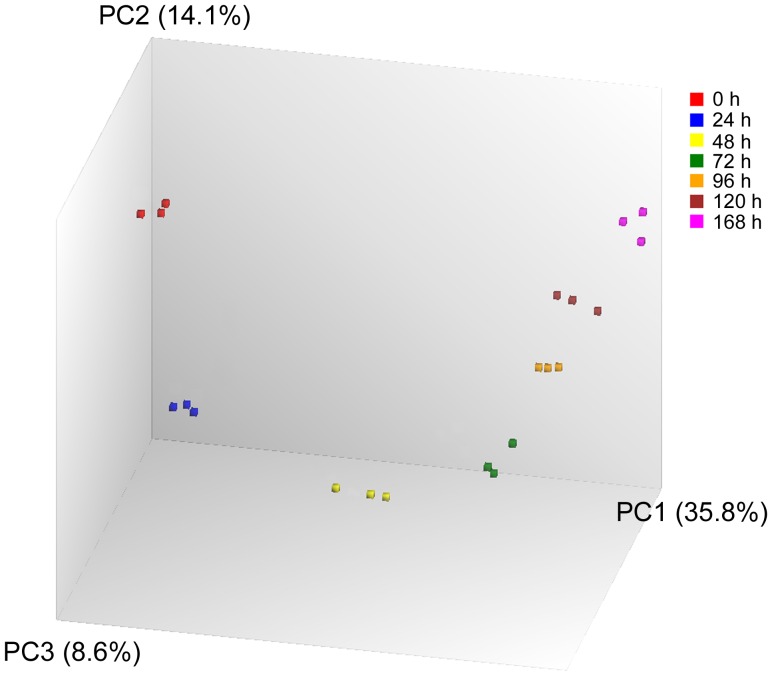
Principal component analysis (PCA) based on all (41,345) probesets in the array to cluster samples based on their similarities or dissimilarities in global gene expression level. The color codes for each time point are shown on the top right corner. The three axes PC1, PC2, and PC3 represent the first three principal components identified by the analysis. The percentage contribution of each component to the overall source of variation is included in the parentheses following each component name.

**Figure 8:**
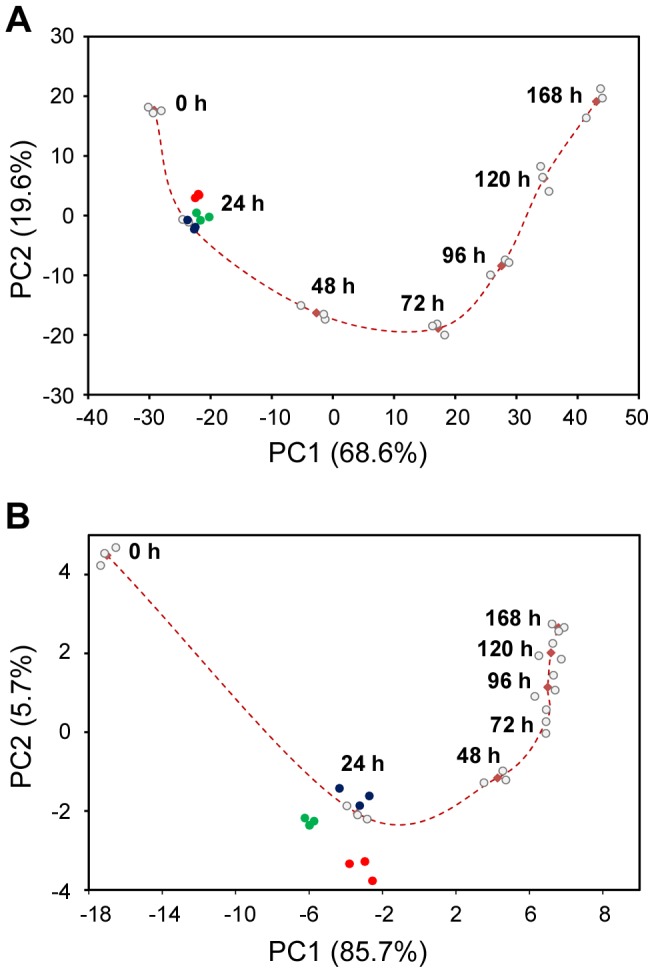
ESC differentiation track shown on two-dimensional PCA plot. The PCA was based (**A**) on the 1996 DEGs, or (**B**) on the 96 DEGs (see text for details on these two gene sets). The control samples at various time points of differentiation are shown as grey dots, and cultures exposed to thalidomide (THD), monobutyl phthalate (MBP), and valproic acid (VPA) for 24 h as blue, green and red dots respectively. The centers for the controls at each time point, defined by their mean values, are shown as dark red squares and the differentiation track is shown as the dashed line connecting the squares.

PCA showed that replicates within each time point group clustered together and different groups appeared in chronological order in the PCA plot (Fig. 7). When all (41,345) probesets on the array were used for the analysis, the variance described by the first and second principal components of the PCA analysis (PC1 and PC2) was only 49.9%. In comparison, when the 1996 DEGs were used for the analysis, the variance described by PC1 and PC2 increased to 88.2%. Further, when only the 96 DEGs were included in the analysis, 91.4% of all variance across all groups at different time points could be described using PC1 and PC2, with the PC1 alone accounting for 85.7% of the variance. The remaining principal components had minor contributions to total gene expression changes and produced no significant shifts between the different time groups. In the two-dimensional PCA plots shown in Fig. 8, a curve connecting the various time point groups can therefore be regarded as a differentiation track delineating gene expression changes during ESC differentiation. And we speculate that developmental toxicants would cause deviation, to a greater or lesser extent depending on their potency, from the differentiation track by affecting gene expression changes during ESC differentiation.

### Compound-Induced Deviation from the Differentiation Track

To verify the hypothesis that developmental toxicants cause deviation from the differentiation track, we examined ESC gene expression changes at 24 h after having been exposed to 0.25 mM thalidomide (THD), 2.0 mM monobutyl phthalate (MBP), or 1.0 mM valproic acid (VPA). Interference of ESC differentiation was studied by analyzing the compound-induced deviation from the differentiation track defined by PCA (Fig. 8). All three compounds showed statistically significant deviations from the differentiation track, but with varying degrees (Table 4). The differentiation cultures exposed to THD deviated statistically significantly only from PC1 of the differentiation track (*p*<0.03), but not from PC2 (*p*>0.05). In comparison, differentiation cultures exposed to MBP and VPA deviated statistically significantly from both principal components. Distances from the center (average) of the exposed cultures to that of the controls also reflect the varying degrees of deviation from the differentiation track, with VPA>MBP>THD. These results suggest that the differentiation track of ESC differentiation as depicted here in the PCA plot can be used to identify differentiation-modulating effects of diverse developmental toxicants.

**Table 4:** Deviation of exposed cultures from the differentiation track based on the 1996 DEGs.

Compound	*p*-value PC1	*p*-value PC2	Distance†
THD	0.021645*	0.058883	0.42
MBP	0.008720**	0.001567**	1.43
VPA	0.001734**	0.000057***	2.64

The comparisons were based on the coordinates (PC1 and PC2) of each sample on the PCA plot.

† Distances from the center (average) of the exposed cultures to that of the controls.

*p<0.05.

**p<0.01.

***p<0.001.

### Compound-Induced Gene Expression Changes in Differentiating ESC

**Figure 9:**
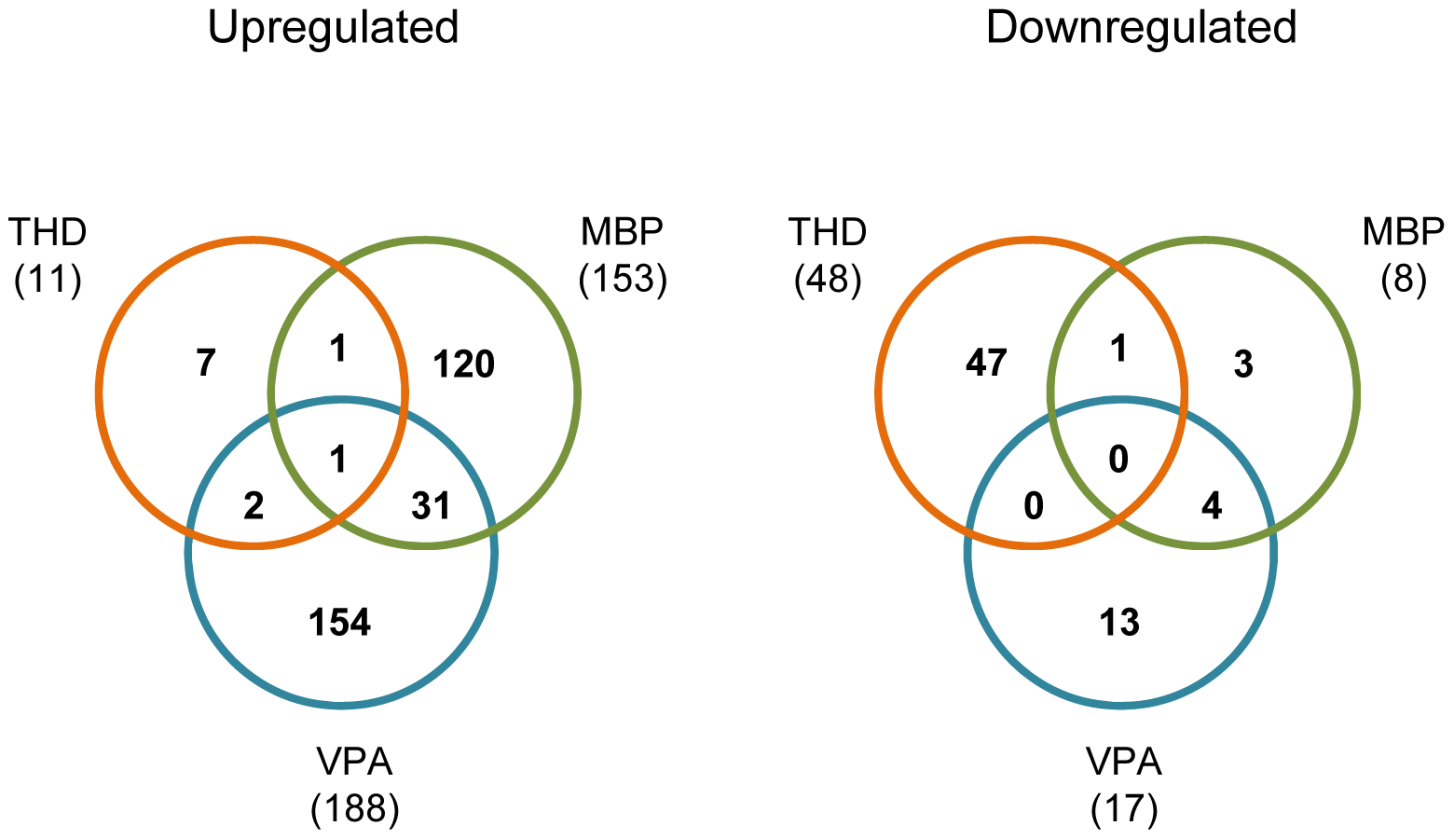
Venn diagrams showing overlap of DEGs induced by different compounds. The total number of upregulated or downregulated DEGs induced by each compound is included in the parentheses under the time. THD, thalidomide; MBP, monobutyl phthalate; VPA, valproic acid.

Comparison of gene expression profiles of differentiation cultures exposed to THD, MBP or VPA with their time-matched controls revealed significant changes in the expression of hundreds of genes (Table 5). Exposure of differentiation cultures to VPA resulted in the greatest number (205) of changed genes, followed by MBP (161) and THD (59). For VPA and MBP, the majority of these DEGs were upregulated, whereas in the case of THD, the number of downregulated DEGs was higher than that of upregulated. Venn diagrams (Fig. 9) show little overlap between compounds as compared to the total numbers of changed genes induced by each compounds.

**Table 5:** Number of DEGs after exposure to THD, MBP or VPA for 24 h compared with their time-matched controls.

Compound	Upregulated	Downregulated	All
THD	11	48	59
MBP	153	8	161
VPA	188	17	205

Pairwise *t*-tests were used for the analysis. The DEGs were selected using *p*<0.05 and FC>2.0.

The 205 DEGs induced by VPA revealed overrepresentation of 14 GO terms (Table S5). These GO terms were grouped into 5 ancestral classes: development (35.7%), morphogenesis (21.4%), reproduction (14.3%), and behavior (7.1%). Similarly, the 161 DEGs induced by MBP led to the enrichment of 6 GO terms (Table S6), with one term (GO:0007275∼multicellular organismal development) belongs to the development class (16.7%). In comparison, the 59 DEGs induced by THD resulted in the enrichment of only one GO term (GO:0009987∼cellular process), which does not belong to any specific ancestral classes.

## Discussion

To date, the best studied model of ESC differentiation is the formation in suspension culture of multicellular aggregates called embryoid bodies (EBs) [37]. Within these aggregates, complex interactions between heterologous cell types result in the induction of differentiation of stem cells to derivatives of all three embryonic germ layers [38]. Plating of the EBs allows further differentiation and EB outgrowth from which cells of endodermal, ectodermal and mesodermal origin were obtained. In EST, pulsating cardiomyocytes were counted at day 10 (7 days after EB formation). Therefore, in the present study, we carried out transcriptomic characterization during a 168 h (7 day) period after EB formation, covering the entire process of ESC differentiation into cardiomyocytes same as in the EST. To our knowledge, this is the most comprehensive study of its kind. A similar but shorter (96 h) study was reported previously by van Dartel *et al.* [39] using D3 mESCs.

Gene expression analysis during the 168 h period of spontaneous ESC differentiation revealed 1996 DEGs. GO analysis revealed these genes were mostly involved in differentiation-related processes, including development, morphogenesis, metabolism, cell differentiation, cell organization and biogenesis, and embryonic development. More specifically, the predominant functions for each of the 8 clusters of these DEGs defined in HCA (Fig. 3) were also closely involved in ESC differentiation (Table 2). For example, tumor suppressor activity, found in 5 of the 8 clusters, plays important role in the prevention of mutations during ESC differentiation [40]. It is interesting to note that the p53 signaling pathway was also enriched by the DEGs (Table 1). An emerging idea in ESC biology is that a p53-dependent pathway may control differentiation, providing an alternative mechanism by which to eliminate damaged cells from the pluripotent stem cell pool. When cells experience oncogenic stress or DNA damage, the p53 protein, a major tumor suppressor, is stabilized and functions to induce transient or permanent cell cycle arrest [41]. Therefore it is not surprising to find that several clusters having functions related to cell cycle (Table 2). Another important function closely related to ESC differentiation is DNA methylation, which is one of the essential epigenetic mechanisms regulating the activation of tissue-specific gene expression during embryonic development. The epigenetic machinery stabilizes the expression of cell type-specific genes and represses genes essential for cell fate decision of unrelated lineages or for maintenance of pluripotency [42]. Adhesion is yet another function worth mentioning. Differential adhesion is implicated in the spreading of one embryonic tissue over another, the sorting out of their cells when intermixed, and the formation of intertissue boundaries respected by the motile border cells [43]. It is further interesting to note several adhesion-related pathways were also enriched by the DEGs, including the focal adhesion pathway, the extracellular matrix (ECM)-receptor interaction pathway, the adherens junction pathway, and the gap junction pathway (Table 1).

Gastrulation is a phase early in the embryonic development of most animals, during which the single-layered blastula is reorganized into a trilaminar structure known as the gastrula. Gastrulation occurs in the following sequence: (1) the embryo becomes asymmetric; (2) the primitive streak forms; (3) cells from the epiblast at the primitive streak undergo an epithelial to mesenchymal transition and ingress at the primitive streak to form the three germ layers known as the ectoderm, mesoderm, and endoderm [44]. Several functions involved in this process were identified in Table 2 in addition to gastrulation *per se*, such as epithelial to mesenchymal transition, mesoderm formation, and paraxial mesoderm formation. It should be noted that in addition to cardiac/heart development, functional analysis of the DEGs (Table 2) revealed some other tissue/organ development during gastrulation, such as axon guidance and neurogenesis (in the ectoderm), chondrogenesis, chondrocyte differentiation and somitogenesis (in the mesoderm). KEGG analysis (Table 1) also revealed pathways involved in specific tissue/organ development in the ectoderm layer, such as the axon guidance pathway and the melanogenesis pathway. Although not on top of the list, endoderm development and several other related terms were identified in the GO terms enriched by the DEGs (Table S2). A marker gene for the hepatic tissue (derived from endoderm), *Alb*, increased its expression rapidly after 120 h of differentiation (Fig. 4). Taking together, these results indicate that development of all three germ layers were included in our model. This is in contrast to the findings of van Dartel *et al.* [39], where only cardiac differentiation was described. The difference may due either to the different cell line used (C57BL/6 *vs.* D3), or to the different duration of the study (168 h *vs.* 96 h). Inclusion in the model of other biological processes in addition to cardiomyocyte differentiation may lead to a broader application domain of the current EST and improved prediction of developmental toxicity.

Inspection of the expression pattern of the marker genes with regard to time (Fig. 4) revealed interesting features of these genes that are in accord with their functions. These patterns generally indicate loss of pluripotency and gain of tissue/organ differentiation. For instance, the ESC specific marker *Tdgf1* and the pluripotency markers *Dppa2*, *Rif1*, and *Zfp42* gradually decreased expression with time, so did the transcriptional factors *Lin28a* and *Utf1*. *Lin28a* is thought to regulate the self-renewal of stem cells [45], and *Utf1* is implicated in maintain stem cell pluripotency, as its expression was found to be rapidly reduced upon differentiation [46]. Our result confirmed this finding. On the other hand, another transcription factor, *Smad3*, increased expression with time. Although *Samd3* was generally considered to regulate cell proliferation, it has been recently found that this gene also plays a critical role in the regulation of ESC differentiation through transcriptional activation [47]. As expected, the markers for early cell types generally increased expression with time, such as those for neural progenitors *Nes*, cardiac progenitors *Isl1* and *Myh6*, early smooth muscle cells *Acta2*, hematopoietic stem cells/early endothelial cells *Tek*, and hepatic tissue *Alb*. It is interesting to note that the expression of *Alb* did not change until at the very late stage. In comparison, expression changes for germ layer markers did not follow the same trend, with some (ectoderm marker *Fgf5* and mesoderm markers *Mixl1* and *T*) decreased with time, while others (ectoderm marker *Meis1* and mesoderm markers *Hand1* and *Bmp4*) increased with time, although *Hand1* and *Bmp4* seemed to have a plateau in the middle and decreased their expression at the later stage. It is also intriguing to note that the expression level of *Eng*, a maker of mesenchymal stem cells, fluctuated during the differentiation process. These patterns may partly be explained by the transient nature of the germ layers and the mesenchymal stem cells. Overall, the results on the expression pattern of the marker genes presented here further support the notion that in our model, gene expression analysis clearly monitored ESC differentiation through the development of all three germ layers. In addition, these results provide useful information regarding the dynamics of gene expression changes of these markers during ESC differentiation, and may spur further studies on the characterization of these genes (and other potential markers of ESC differentiation) at the protein and cellular level.

The ESC differentiation process was analyzed by performing PCA on the dataset using either the 1996 DEGs or the 96 DEGs, which both showed a continuous PCA trajectory that defines the differentiation track. Mathematically, the 1996 DEGs could be considered as the product of “union” of the DEGs at all the time points, whereas the 96 DEGs (annotated genes from the 132 DEGs shown in Fig. 5) could be visualized as the product of “intersection” of the DEGs at each the time point. The 1996 DEGs were found to be fairly uniformly dispersed among the various time points (data not shown), and as a consequence, the differentiation track defined by the 1996 DEGs was a faithful description of the entire differentiation process covered in this study (0–168 h) (Fig. 8A). In contrast, the differentiation track defined by the 96 DEGs, although having a slighter better accuracy than the one defined using the 1996 DEGs in describing the variance in the dataset (91.4% *vs.* 88.2%), was nevertheless heavily skewed, with the first few time points (around 24 h) widely separated whereas the later time points condensed considerably. The reason lies in the fact that the 132 DEGs, from which the 96 DEGs were derived, accounts for >34% of all DEGs at 24 h (386), but the percentage dropped to <14% for 48 h (974), <10% for 72 h (1427), 96 h (1397), and 120 h (1567), and only ∼6% for 168 h (2122). Therefore, the 96 DEGs placed a larger weight on 24 h than on the later time points, hence a better separation at this time point. This is readily reflected in Fig. 8B, where the compound-exposed samples at 24 h had a better separation from the control samples than in Fig. 8A.

The differentiation track described here could be employed as a baseline for developmental toxicity testing. One feasible way to do this is by calculating the deviation of compound-exposed cultures from the differentiation track, as shown in Table 4. The statistics showed that all three compounds deviated significantly from the differentiation track, although the deviation by THD was only on one axis (PC1). An inherent problem when comparing toxicogenomic effects of different compounds is that the exposure levels may not be equivalent. However, at the doses tested here, both the degrees of significance and the distances between the center of compound exposed cultures and that of the vehicle controls suggest that VPA had the greatest potency among the three in modulating ESC differentiation, while THD is the least potent, as supported by the number of total DEGs induced by each compound (Table 5). The method described here may be further exploited in the future to classify compounds into different categories of developmental toxicity as non-toxic, weakly toxic, moderately toxic, and strongly toxic. A list of compounds with known development toxicities can be used to train the model and to define the grade values for subsequently stratifying unknown potential developmental toxicants.

The fact that the three compounds tested here shared little common genes as compared to the total numbers of changed genes induced by each compound (Fig. 9) suggest that, in accordance with previous findings [23]–[25], these compounds exert their developmental actions by different mechanisms of action. In particular, THD was distinct from VPA and MBP in that for THD, the number of downregulated DEGs was higher than that of upregulated, whereas for VPA and MBP, the majority of these DEGs were upregulated. In addition, THD had far less total DEGs (59) than VPA (205) and MBP (161). For both VPA and MBP, BP GO terms in the class of development were enriched on top of the list; in contrast, no GO term enrichment was found for THD. This is perhaps not surprising as THD is not teratogenic in some animal species, particularly the mouse [23]. One possibility is that thalidomide does not pass through the mouse placenta. Alternatively, the antiangiogenic metabolic products of THD are not generated in these species [48]. Nevertheless, the perturbing effect on gene expression by direct exposure to THD was still detectable in the present model, albeit to a less extent than VPA and MBP. Taking together, these results further support the notion that by inclusion in our model of other biological processes in addition to cardiomyocyte differentiation, the application domain of the model was broadened so as to be capable of identifying differentiation-modulating effects of diverse developmental toxicants.

The findings presented here are encouraging in that we have not only demonstrated the feasibility of incorporating a group of genes (1996 DEGs) to the EST model to potentially broaden its application domain and improve its prediction, we have also expanded the repertoire of mESC lines that could potentially be employed in the EST. Although several germline-competent mESCs have been derived from C57BL/6 mice in the past decade [20], [21], to our knowledge none of them have been characterized transcriptomically. More importantly, using C57BL/6 mESCs in our model we were able to identify the development of many different cell types in all three germ layers that were not reported previously using a D3 cell line [39]. Thus the current study represents the first in its kind and would spur further studies along the same line.

Finally, it should be noted that the current model has its limitations in that although gene expression analysis clearly monitored ESC differentiation into multiple lineages in all three germ layers, it is not possible to depict in this simple *in vitro* model all the complex interactions between cells, tissues and organs occurred during embryonic development *in vivo*. Therefore, more research is needed before it could ultimately serve as an alternative model for developmental toxicity testing.

## Conclusions

In this study, as part of an effort to develop an ESC-based alternative model for the assessment of developmental toxicity, we characterized gene expression changes during the differentiation of a C57BL/6-derived mESC cell line, which covered a period of 168 h (7 d) after EB formation. To our knowledge, this is the most comprehensive transcriptomic characterization of mESC differentiation, and the first one using a C57BL/6 strain.

We showed that the overall gene expression profiles of the C57BL/6 mESCs gradually changed during the course of differentiation, and 1996 genes were found to be differentially expressed during the differentiation process. This gene set covers the development of all three germ layers or that of their derivative early cell types. Incorporation of this gene set in the EST model may lead to a broader application domain of the current EST and improved prediction of developmental toxicity.

Using this gene set, we defined a differentiation track on the two-dimensional PCA plot. We demonstrated the differentiation track was capable of identifying the modulating effects of diverse developmental toxicants. We propose that the differentiation track defined in this study be further exploited as a baseline for developmental toxicity testing, with compounds causing significant deviation from the differentiation track being predicted as potential developmental toxicants.

## Supporting Information

Table S1 **Full list of DEGs across different time points during ESC differentiation.** Using stringent criteria of Bonferroni-adjusted *p*<0.05 and fold change (FC)>2.0, a total of 1996 DEGs were found differentially expressed, with 1229 upregulated and 767 downregulated along the time course. Out of these probesets, 1675 were mapped to the DAVID database as annotated genes.
(XLSX)

Table S2 **Full list of 541 (533 unique) GO terms in the biological process (BP) category at all levels (GOTERM_BP_ALL) enriched by the 1996 DEGs identified during ESC differentiation.** DAVID was used for the analysis using the 1996 DEGs identified during ESC differentiation (see text and Table S1 for details). The *Mus musculus* (mouse) whole genome was used as background. Statistical enrichment was determined through a modified Fisher's exact test (*p*<0.05) and count threshold >4. The column under “Count” indicates the number of DEGs involved in the specific GO term, while the column under “%” indicates the number of DEGs involved in the GO term as a percentage of the total number of annotated DEGs (1675).
(XLSX)

Table S3 **List of 96 DEGs that were differentially expressed throughout the ESC differentiation process.** Gene expression profiles at each time point were compared with the starting point (0 h after EB formation) using pairwise *t*-test, and DEGs were selected by *p*<0.05 and FC>2.0. The common DEGs of all the time points were mapped to DAVID database to find the annotated genes.
(XLSX)

Table S4 **Full list of 162 (159 unique) GO terms in the biological process (BP) category at all levels (GOTERM_BP_ALL) enriched by the 96 DEGs identified throughout ESC differentiation.** DAVID was used for the analysis using the 96 annotated genes differentially expressed throughout ESC differentiation (see text and Table S3 for details). The *Mus musculus* (mouse) whole genome was used as background. Statistical enrichment was determined through a modified Fisher's exact test (*p*<0.05) and count threshold >4. The column under “Count” indicates the number of DEGs involved in the specific GO term, while the column under “%” indicates the number of DEGs involved in the GO term as a percentage of the total number of DEGs (96).
(XLSX)

Table S5 **List of 16 GO terms in the biological process (BP) category at all levels (GOTERM_BP_ALL) enriched by the 205 DEGs induced by VPA exposure.** DAVID was used for the analysis using 57 unique DAVID IDs (annotated genes) mapped from the 205 DEGs induced by VPA. The *Mus musculus* (mouse) whole genome was used as background. Statistical enrichment was determined through a modified Fisher's exact test (*p*<0.05) and count threshold >4. The column under “Count” indicates the number of DEGs involved in the specific GO term, while the column under “%” indicates the number of DEGs involved in the GO term as a percentage of the total number of annotated genes (57).
(XLSX)

Table S6 **List of 6 GO terms in the biological process (BP) category at all levels (GOTERM_BP_ALL) enriched by the 161 DEGs induced by MBP exposure.** DAVID was used for the analysis using 8 unique DAVID IDs (annotated genes) mapped from the 161 DEGs induced by VPA. The *Mus musculus* (mouse) whole genome was used as background. Statistical enrichment was determined through a modified Fisher's exact test (*p*<0.05) and count threshold >4. The column under “Count” indicates the number of DEGs involved in the specific GO term, while the column under “%” indicates the number of DEGs involved in the GO term as a percentage of the total number of annotated genes (8).
(XLSX)
